# Urinary Vanin-1 in the detection of acute kidney injury in humans

**DOI:** 10.1186/s12882-026-05002-z

**Published:** 2026-05-28

**Authors:** Konstantinos Markakis, Panagiota Zgoura, Maximilian Seidel, Jonas Franz Kolodziej, Sonja Rieckmann, Sebastian Bertram, Adrian Doevelaar, Benjamin Rohn, Nina Babel, Timm Westhoff, Felix Seibert

**Affiliations:** 1 Medical Department I, University Hospital Marien Hospital Herne, Ruhr-University of Bochum, Herne, Germany; 2 First Department of Internal Medicine, AHEPA University General Hospital, Aristotle University of Thessaloniki, Stilponos Kyriakidi 1, Thessaloniki, 54636 Greece

**Keywords:** Vanin-1, Acute kidney injury, Prerenal, Intrinsic, Biomarker

## Abstract

**Background:**

Former studies indicate, that urinary Vascular Non-Inflammatory Molecule 1 (Vanin-1) may be an early biomarker of acute tubular necrosis. We evaluated the diagnostic performance of Vanin-1 regarding the detection of acute kidney injury (AKI) and the differentiation of prerenal from intrinsic AKI compared to other known biomarkers.

**Materials and methods:**

Urinary Vanin-1, Neutrophil gelatinase-associated Lipocalin (NGAL), Kidney injury molecule 1 (KIM-1) and calprotectin concentrations were assessed in 112 hospitalized subjects, 51 with intrinsic and 61 with prerenal AKI. 27 healthy subjects served as controls. Exclusion criteria were postrenal AKI or renal transplantation. Only patients with severe AKI were eligible. Results were expressed as urinary creatinine ratios.

**Results:**

Median urinary Vanin-1/creatinine was significantly higher in patients with AKI (1912 [812.1–4808] vs. 344.2 [165.2-553.4] pg/mg, *p* = 0.001; AUROC 0.86 [95% CI 0.80–0.93], *p* = 0.001). The AUROC for NGAL-, KIM-1- and calprotectin/creatinine were 0.92 (95% CI 0.87–0.97; *p* = 0.001), 0.96 (95% CI 0.92–0.99; *p* = 0.001), 0.82 (95% CI 0.74–0.90; *p* = 0.001), respectively. The diagnostic accuracy of urinary Vanin-1/creatinine in the differentiation of prerenal from intrinsic AKI was low (AUROC 0.64 [95% CI 0.54–0.75, *p* = 0.01]). The respective AUROCs for NGAL/creatinine, calprotectin/creatinine and KIM-1/creatinine were 0.71 ([95% CI 0.61–0.80], *p* = 0.001), 0.77 ([95% CI 0.68–0.86], *p* = 0.001) and 0.54 ([95% CI 0.44–0.65], *p* = 0.001).

**Conclusion:**

To our knowledge, this is one of the largest human studies evaluating urinary Vanin-1 in AKI. Urinary Vanin-1/creatinine ratio was higher in patients with AKI compared to controls. However, its diagnostic accuracy in the differentiation of intrinsic from prerenal AKI was lower than of other biomarkers.

## Introduction

Acute kidney injury (AKI) occurs in up to 22% of hospitalized persons and is associated with the prognosis of the kidney function [1]. In the last decade, a multitude of studies have focused on early detection and differentiation between the different categories of AKI, in particular between prerenal and intrinsic AKI. In many cases, after ruling out postrenal causes, the question arises as to whether the AKI is of prerenal or intrinsic aetiology. Relying on simple urine analysis and on parameters like creatinine (sCr), urea and urine output for differentiating between prerenal and intrinsic AKI often proves to be inadequate for establishing the correct diagnosis. As a result, the risk of misdiagnosis and of a suboptimal or even hazardous therapeutic approach increases. This in turn, increases the risk of AKI transitioning into acute kidney disease (AKD), an intermediary stage with on-going pathophysiological processes which can end in chronic kidney disease (CKD) [2]. Furthermore, a certain overlap between prerenal and intrinsic AKI exists. In particular, prerenal AKI can evolve into acute tubular necrosis (ATN) if not treated properly and consequently those two conditions should rather be considered a continuum than two separate entities [3]. To make matters worse pre-existing CKD can make the interpretation of urine analysis and laboratory parameters difficult rendering a correct diagnosis challenging [4]. To overcome these diagnostic challenges, the capacity of several biomarkers to differentiate between prerenal and ATN has been evaluated in the past. Three of the most studied biomarkers include the Neutrophil gelatinase-associated Lipocalin (NGAL), the urinary calprotectin and the Kidney injury molecule 1 (KIM-1). Initial reports supported the diagnostic ability of those parameters to adequately distinguish prerenal from intrinsic AKI [5]. Nonetheless, subsequent studies were less supportive [6]. Our studies regarding urinary calprotectin showed a certain diagnostic potential, which could help a clinician to decide for or against a renal biopsy [7, 8].

In the search for novel renal biomarkers, that could achieve the transition from bench to bedside, we investigated the Vascular Non-Inflammatory Molecule 1 (Vanin-1), also known as pantetheinase, a 60–80 kDa monomeric glyco-protein [9]. It is expressed in a variety of organs including kidneys and hydrolyzes pantetheine into cysteamine and pantothenic acid, a precursor of coenzyme A [10, 11]. Cysteamine is subsequently turned into cystamine, an important factor in the regulation of glutathione production-rate. In vanin-knockout mice, the resulting lack of tissue-cysteamine renders them resistant to oxidative stress and mitigates the stress-induced damage [12]. Recent studies in animal models indicate that urinary Vanin-1 may be used as a biomarker for the early detection of tubular damage caused by renal ischemia or nephrotoxic agents [13–15]. Kellum et al. categorized the biomarkers under evaluation into two main groups, namely functional and damage biomarkers [16]. The first ones identify patients at increased risk of developing AKI while those in the second group diagnose AKI by documenting kidney damage. A subsequent work proposed a third group of biomarkers consisting by molecules released by inflammatory cells during AKI, like IL-18 [17]. Vanin-1 would be placed on the second group as it is characterized by increased release in AKI and is mainly associated with tubular damage. Consistent with previous findings showcasing Vanin-1’s role in glutathione production, Hosohata et al., observed that in Dahl sensitive mice, a high salt-intake resulted in a rise of reactive oxygen species and in renal tubular damage. When those effects were lifted, a decrease of vanin-1 levels was observed [13]. Furthermore, in animals treated with ethylenoglycol an increase of vanin-1 but not of monocyte chemoattractant protein 1 (MCP-1) and NGAL was observed. In an in-vitro study of human renal cells a more rapid increase of vanin-1 mRNA levels than of MCP-1 and KIM-1 was witnessed after exposure to nephrotoxicants [15]. Lastly, in animal models subjected to experimental renal ischemia and then reperfusion, a decline of vanin-1 levels was observed when the ischemia was lifted [18]. Based on these findings, we hypothesized that urinary Vanin-1 could help in the assessment of AKI in hospitalized patients. This study investigates the discriminatory potential of Vanin-1 in AKI and its capacity to differentiate between prerenal and intrinsic AKI.

## Materials and methods

### Study population

The study protocol was similar to our former studies [7, 8]. A cross-sectional study at the Charité – University Hospital Berlin and at the University Hospital Marien Hospital Herne was performed. The study was approved by the local ethics committee of the Charité - University Hospital Berlin (EA4/016/12) and by the ethics committee of the Ruhr-University of Bochum (5019-14**)**. All participants provided written informed consent.

Hospitalized patients with AKI of unknown origin were enrolled in the study. AKI was defined according to the Kidney Disease: Improving Global Outcomes (KDIGO) criteria [19].

Inclusion criteria were:

- The presence of severe AKI according to the KDIGO criteria (Stage II, Stage III).
- Age > 18 years old.

Exclusion criteria were:

- Kidney transplant recipients.
- Obstructive uropathy.
- Gravidity.

The study population consisted of patients who were hemodynamically stable and did not require any invasive monitoring or vasopressor, hence were admitted to the internal medicine ward.

Epidemiological data, laboratory parameters, origin of AKI, concomitant diseases and premedication of the participants were recorded. The laboratory parameters used in this study were recorded in the day of the urine samples collection. Preexisting CKD was defined according to KDIGO criteria. Performing a kidney biopsy and a histological examination was the gold standard method for differentiating between prerenal and intrinsic AKI. Patients with acute or chronic cardiorenal (Type I and II according to the American Heart Association) with Type I or Type II hepatorenal syndrome, as well as bilateral renal artery stenosis, were categorized as cases of prerenal AKI, as the driving pathophysiological mechanism in these cases was the reduced effective renal arterial flow. In those subjects where no kidney biopsy was performed and which also did not have any of the above conditions, the diagnosis was established by applying a prepublished clinical algorithm [7] (Table 1). According to this, a rapid response of renal function to volume repletion is defined as an obligatory criterion (category A) for the diagnosis of prerenal AKI. Balanced crystalloid solutions were used for volume repletion. A reconstitution of the renal function to at least 80% of the initial, pro-AKI, glomerular filtration rate (GFR) in the first 48 h was regarded as a positive response to volume repletion. Compatibility of history, physical findings and urinary findings (absence of proteinuria, haematuria and pyuria) are regarded as category B criteria. Two criteria of category B and the category A criterion are required to establish the diagnosis for prerenal AKI. Hepatorenal and cardiorenal syndrome, as well as bilateral renal artery stenosis were, independently regarded prerenal AKI.

**Table 1:** Diagnostic criteria for differentiating prerenal from intrinsic AKI

Clinical Criteria	Prerenal AKI	Intrinsic AKI
**Category A**		
response to volume repletion	Rapid decrease of creatinine with convergence to baseline levels	No reconstitution of renal function
**Category B**		
a) history compatible	Dehydration, loss of fluid, e.g., by gastroenteritis, heart failure, liver failure, inadequate use of diuretics, etc.	Prolonged shock, exposition to nephrotoxins, extrarenal suggestive symptoms like pulmorenal syndrome, etc.
b)physical findings compatible	Low blood pressure, low jugular pulse, tachycardia, orthostatic changes, poor skin turgor	Absence of signs of dehydration, cardiac monitoring shows adequate volemia
c) urine examinations	Absence of proteinuria, hematuria, and leukocyturia	Proteinuria and/or hematuria and/or leukocyturia

category A: obligatory, criteria B: optional for the diagnosis of prerenal or intrinsic AKI. AKI: acute kidney injury

### Measurement of Vanin-1, calprotectin, NGAL, KIM-1

Urine samples (10 ml) were collected five times weekly at our nephrological ward in an effort to keep the time period from the initial diagnosis of AKI as short as possible and ≤ 3 days. The samples were stored frozen (− 20 °C) until assessment of Vanin-1 concentration took place. Urine concentrations of Vanin-1 were quantified using an ELISA kit (Human Vanin-1 (urine) ELISA, catalog-number BI-Van1U; Biomedica Mediziniprodukte GmbH, Vienna, Austria) according to the manufacturer’s protocol. The lower limit of detection (LOD) for this ELISA kit is 9.6 pmol/l and the lower limit of quantification (LLOQ) 38 pmol/l. The intraassay coefficient of variation (CV) is ≤ 5% and the interassay CV ≤ 7%. Respectively, the urine concentrations of calprotectin and NGAL were quantified using an ELISA kit (PhiCal^®^ Calprotectin, catalog number K 6935; Immundiagnostik AG, Bensheim, Germany) according to manufacturer’s constructions. Urine NGAL concentrations were assessed with an ELISA kit as well (NGAL kit 037; Bioporto, Gentofte, Denmark). To take the current concentration status of the urine into account, urinary creatinine levels were measured and calprotectin/creatinine relations calculated. NGAL, KIM-1 and urinary Vanin-1 concentrations were expressed as ratios to the respective urinary creatinine as well. The investigator performing the ELISA measurements was blinded for clinical data. Urine concentrations of calprotectin were quantified using an enzyme-linked immunosorbent assay (ELISA) kit (PhiCal^®^ Calprotectin, catalogue number K 6935; Immundiagnostik AG, Bensheim, Germany) according to the manufacturer’s protocol. Similarly, the concentrations of NGAL and KIM-1 were assessed using ELISA as well (NGAL kit 037; Bioporto, Gentofte, Denmark and ADI-900-226-0001 from Enzo Life Science, respectively).

The transition from prerenal AKI to ATN represents a continuum. As a dynamic process, changes in kidney function and laboratory parameters may occur even within the same day, rendering correct AKI classification and validation of AKI biomarkers difficult. To minimize the impact of time and avoid misclassification of AKI, in cases were a kidney biopsy was performed effort has been made to keep the interval between urine samples collection and kidney biopsy as short as possible. Hence, urine collection was restricted in the last 24 h before renal biopsy was planned.

### Statistics

Data were analysed for Gaussian distribution (D’Agostino Pearson). In case of normal distribution, data were presented as mean with standard deviation (SD), otherwise, n as median with the respective interquartile range (IQR). Differences between groups were assessed using Wilcoxon rank sum test. Receiver-operating characteristic (ROC) curves were formed in an attempt to determine the accuracy of urinary Vanin-1 in predicting an intrinsic cause of AKI. ROC curves and Youden index were used to establish the best cut-off value in this differentiation. Sensitivity, specificity, positive and negative predictive value were calculated using this threshold. The numeric parameters of the participants with prerenal or intrinsic AKI were compared by applying the two-sided unpaired Student’s t-test. Categorical parameters were compared with Fisher’s exact test in case of dichotomy (presence/absence of concomitant diseases, medication, biopsy, gender) and with Pearson chi-square test in case of polychotomy (stage of AKI). After an initial statistical analysis of the present study population, procedures were repeated for the combination of the populations of the proof of concept trial and the current study. A p-value of < 0.05 was regarded as statistically significant. P values were not corrected for multiple testing, as this study was of exploratory nature [20, 21]. Patients with indeterminate or missing data were excluded from the study. However, no such case among the eligible patients occured. All statistical analyses were performed using GraphPad Prism (GraphPad Software, California, USA) and IBM SPSS Statistics version 26 (IBM Corp., Armonk, N.Y., USA).

## Results

### Epidemiological data

**Fig. 1:**
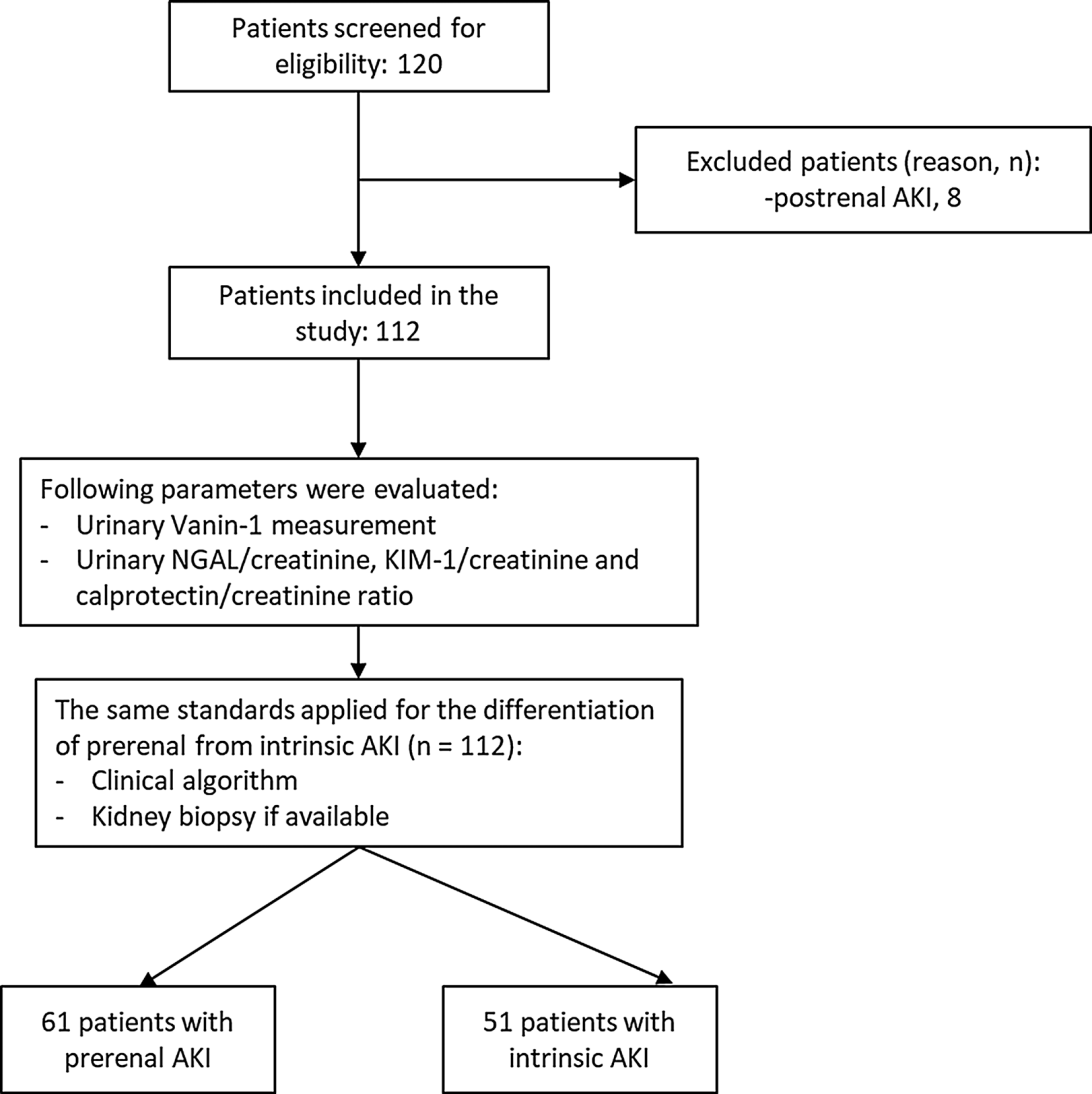
Study design. Abbreviations: AKI: acute kidney injury

139 subjects were enrolled in the study, which included 112 hospitalized subjects with AKI and 27 subjects without medical history which served as controls. Among those patients with AKI, 61 cases were diagnosed with prerenal AKI according to the aforementioned clinical criteria and classification, while 51 patients with intrinsic AKI (Fig. 1).

The epidemiological data, cause of AKI, medical history, premedication and the KDIGO stage of the participants with prerenal, intrinsic or normal renal function were compared (Table 2). Compared to the persons serving as controls, patients with AKI were significantly older and had significantly more often a history of arterial hypertension, diabetes mellitus, hyperlipidemia or smoking (*p* = 0.001). Consequently, they had been treated significantly more often with diuretics, angiotensin converting enzyme–inhibitors (ACE-I) or angiotensin II receptor blockers (ARB) than controls (*p* = 0.001). Male/Female ratio and body mass index (BMI) were similar between the two groups.

**Table 2:** Epidemiological data

	Total study population (*n* = 139)	AKI (*n* = 112)	No AKI (*n* = 27)	*p*	prerenal AKI (*n* = 61)	intrinsic AKI (*n* = 51)	*p*
Female; Male	53 (38.1%); 86 (61.9%)	44 (31.7%); 68 (48.9%)	9 (6.5%); 18 (12.9%)	0.568	18 (12.9%); 42 (30.2%)	20 (17.9%); 31 (27.7%)	0.054
Age (years)	67 [51–75]	70 [54–77]	44 [27–65]	**0.001**	63 [54–74]	67 [57–79]	0.091
Body mass index	26.3 [23.2–28.1]	26.4 [23.1–28.2]	25.9 [21.9–27.7]	0.538	26.4 [26.6–28.9]	26.7 [23.1–28.2]	0.838
KDIGO stage II	30/112	30 (26.8%)	-	**0.001**	19 (17.0%)	11 (9.8%)	0.254
KDIGO stage III	82/112	82 (73.2%)	-		42 (37.5%)	40 (35.7%)	
Creatinine (mg/dl)		2.4 (IQR 2.7)	1 (IQR 0)	**0.001**	2 (IQR 2.54)	2.8 (IQR 2.3)	**< 0.01**
Urea (mg/dl)		65 (IQR 46.7)			70 (IQR 46.6)	56.3 (IQR 35)	**< 0.01**
Hypertension	79 (56.8%)	74 (53.2%)	5 (3.6%)	**0.001**	43 (38.4%)	31 (27.7%)	0.280
Diabetes	26 (18.7%)	23 (16.5%)	3 (2.2%)	0.407	14 (12.5%)	9 (8.0%)	0.489
Coronary heart disease	25 (18.0%)	24 (17.3%)	1 (0.7%)	0.177	18 (16.1%)	6 (5.4%)	**0.023**
Hyperlipidemia	29 (20.9%)	27 (19.4%)	2 (1.4%)	0.410	15 (13.4%)	12 (10.7%)	0.896
Smoking history	27 (19.4%)	27 (19.4%)	0 (0.0%)	**0.038**	19 (17.0%)	8 (7.1%)	0.057
ACE-I or ARB	41 (29.5%)	37 (26.6%)	4 (2.9%)	0.052	27 (24.1%)	10 (8.9%)	**0.005**
Diuretics	50 (36.0%)	49 (35.3%)	1 (0.7%)	**0.001**	31 (27.7%)	18 (16.1%)	0.099
MRB	6 (4.3%)	4 (2.9%)	0 (0.0%)	0.319	4 (3.6%)	0 (0.0%)	0.063

ACE-I: angiotensin converting enzyme inhibitor, ARB: angiotensin II receptor blocker, IQR: interquartile range, MRB: mineralcorticoid receptor blocker

Among subjects with prerenal AKI, 19 (31.1%) patients were classified as KDIGO stage II while 42 (68.9%) as KDIGO stage III. Among patients with intrinsic AKI, 11 (21.6%) presented with KDIGO stage II, while 40 (78.4%) with stage III. In 21 patients with AKI a kidney biopsy was performed (18.75%). The AKI-stage, measured according to the KDIGO criteria, between patients with prerenal and patients with intrinsic AKI didn’t differ (*p* = 0.254). Moreover, no statistically significant difference between the two groups, regarding sex, age, BMI has been recorded. As to the medical preconditions, patients with prerenal AKI had significantly more frequent history of coronary heart disease than those with intrinsic AKI (16.1% vs. 5.4%, *p* = 0.023). Prevalence of arterial hypertension (*p* = 0.28), diabetes (*p* = 0.489) and hyperlipidemia (*p* = 0.896) did not differ between the two groups. Patients who presented with prerenal AKI had been treated significantly more often with ACE-I or ARB than patients with intrinsic AKI (24.1% vs. 8.9%, *p* = 0.005). Previous treatment with mineralocorticoid receptor blockers (MRB), was more frequent in patients with prerenal AKI than in participants with intrinsic AKI (3.6% vs. 0%). Nevertheless, this difference did not reach statistical significance (*p* = 0.063). Interestingly, treatment with diuretics did not differ between patients with prerenal and intrinsic AKI (27.7% vs. 16.1%, *p* = 0.099). Lastly, sCr and urea were significantly higher in patients with prerenal than with intrinsic AKI (*p* < 0.01) (Table 2).

### Causes of AKI

The various, different underlying, causative conditions of AKI for each patient have been recorded and are demonstrated in Table 3. Multiple conditions associated with AKI can be present at each participant. In patients where no kidney-biopsy was performed, the differentiation between prerenal and intrinsic AKI has been based on the clinical algorithm mentioned previously. Dehydration and hypotension were recorded as the primary causes of AKI significantly more frequently among patients classified as having a prerenal AKI, than in patients with intrinsic AKI [dehydration: 54 (88.5%) vs. 23 (45.1%), *p* < 0.001; hypotension 32 (52.5%) vs. 10 (19.6%), *p* < 0.001]. Other causes of dehydration, except from gastroenteritis, were significantly more frequent in participants with prerenal than with intrinsic AKI [20 (32.8%) vs. 0, *p* < 0.001]. AKI caused by circulatory shock, toxic agents or in the context of pulmonorenal syndrome was more common among patients with intrinsic than in participants with prerenal AKI [27 (52.9%) vs. 14 (23%), *p* = 0.001]. Likewise, drug-induced acute interstitial nephritis and multiple myeloma were present in more patients with intrinsic than with prerenal AKI [interstitial nephritis: 17 (33.3%) vs. 2 (3.3%), *p* < 0.001; multiple myeloma: 5 (9.8%) vs. 0, *p* = 0.01]. As expected, a pathological urine sample examination, defined as the presence of hematuria or proteinuria, was found significantly more often in patients with intrinsic AKI than those with prerenal AKI [41 (80.4%) vs. 28 (45.9%), *p* < 0.001].

**Table 3:** Causes of AKI

	Prerenal AKI (*n* = 61)	Intrinsic AKI (*n* = 51)	*P*
Dehydration by gastroenteritis	54 (88.5%)	23 (45.1%)	< 0.001
Hypotension (hemodynamic, drug-induced and others)	39 (63.9%)	12 (23.5%)	**< 0.001**
Toxins, Shock or pulmorenal-syndrome	14 (23%)	27 (52.9%)	**0.001**
Pathological urine (proteinuria, hematuria, leukocyturia or presence of crystals)	28 (45.9%)	41 (80.4%)	**< 0.001**
Other causes of dehydration	20 (32.8%)	0	**< 0.001**
Cardiorenal syndrome	6 (9.8%)	2 (3.9%)	0.23
Hepatorenal syndrome	3 (4.9%)	1 (2%)	0.41
Bilateral renal artery stenosis	2 (3.3%)	0	0.19
Hypotension-induced acute tubular necrosis	2 (3.3%)	0	0.19
Toxic acute tubular necrosis	0	3 (5.9%)	0.054
Crescentic glomerulonephritis	0	1 (2%)	0.27
Thrombotic microangiopathy (TTP, aHUS)	1 (1.6%)	4 (7.8%)	0.11
Drug-induced acute interstitial nephritis	2 (3.3%)	17 (33.3%)	**< 0.001**
Virus-associated nephropathy	0	3 (5.9%)	0.054
Rhabdomyolysis	3 (4.9%)	2 (3.9%)	0.8
Urosepsis/severe urinary tract infection	9 (14.8%)	12 (23.5%)	0.24
Contrast-media induced	0	1 (2%)	0.27
Multiple myeloma	0	5 (9.8%)	**0.01**
Unknown	2 (3.3%)	1 (2%)	0.4

aHUS: atypical haemolytic uremic syndrome, AKI: acute kidney injury, ACE-I: angiotensin converting enzyme inhibitor, ARB: angiotensin II receptor blocker, NSAID: non-steroidal anti-inflammatory drugs, TTP: thrombotic thrombopenic purpura

### Performance of Vanin-1 as a discriminatory marker

**Fig. 2:**
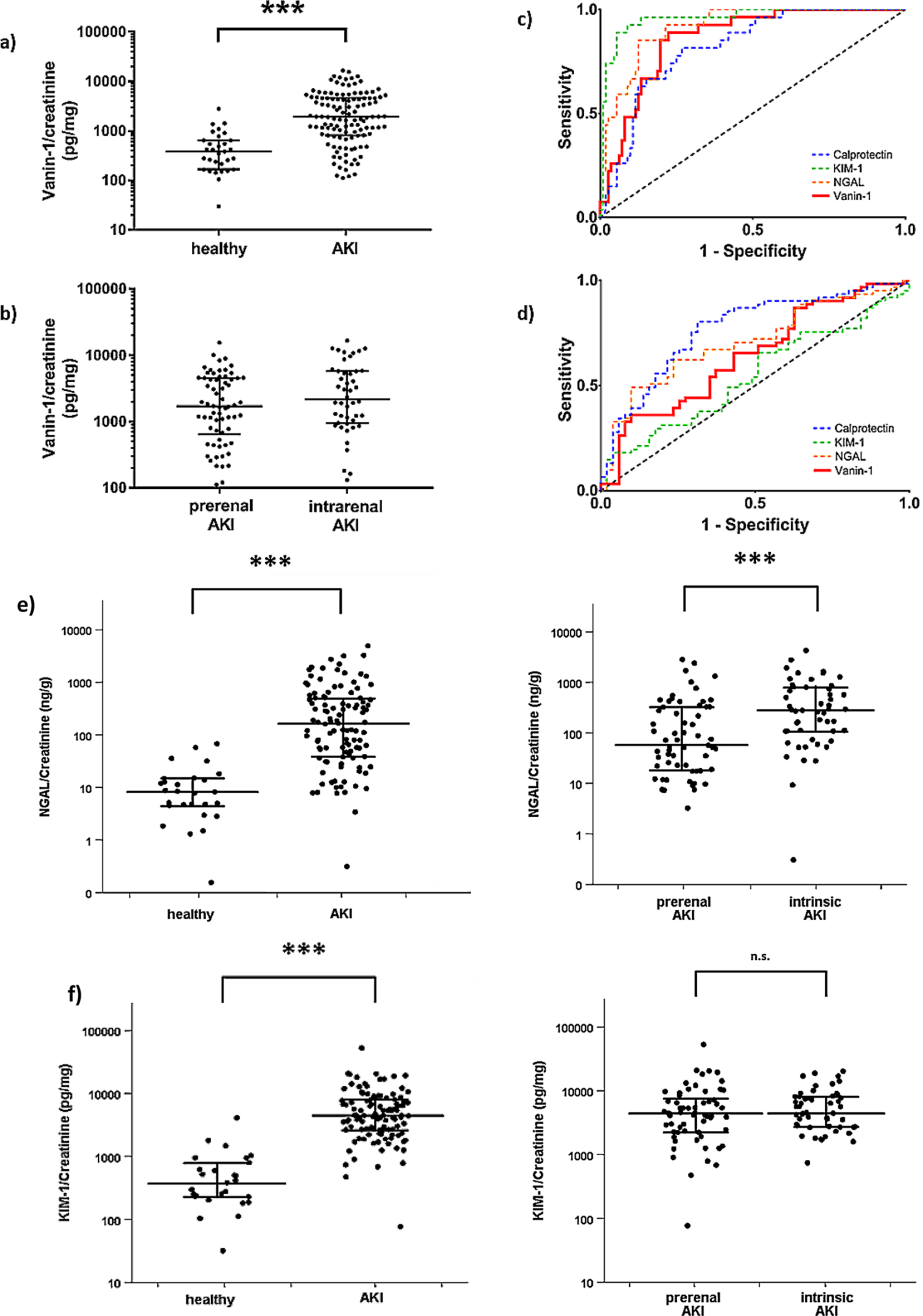

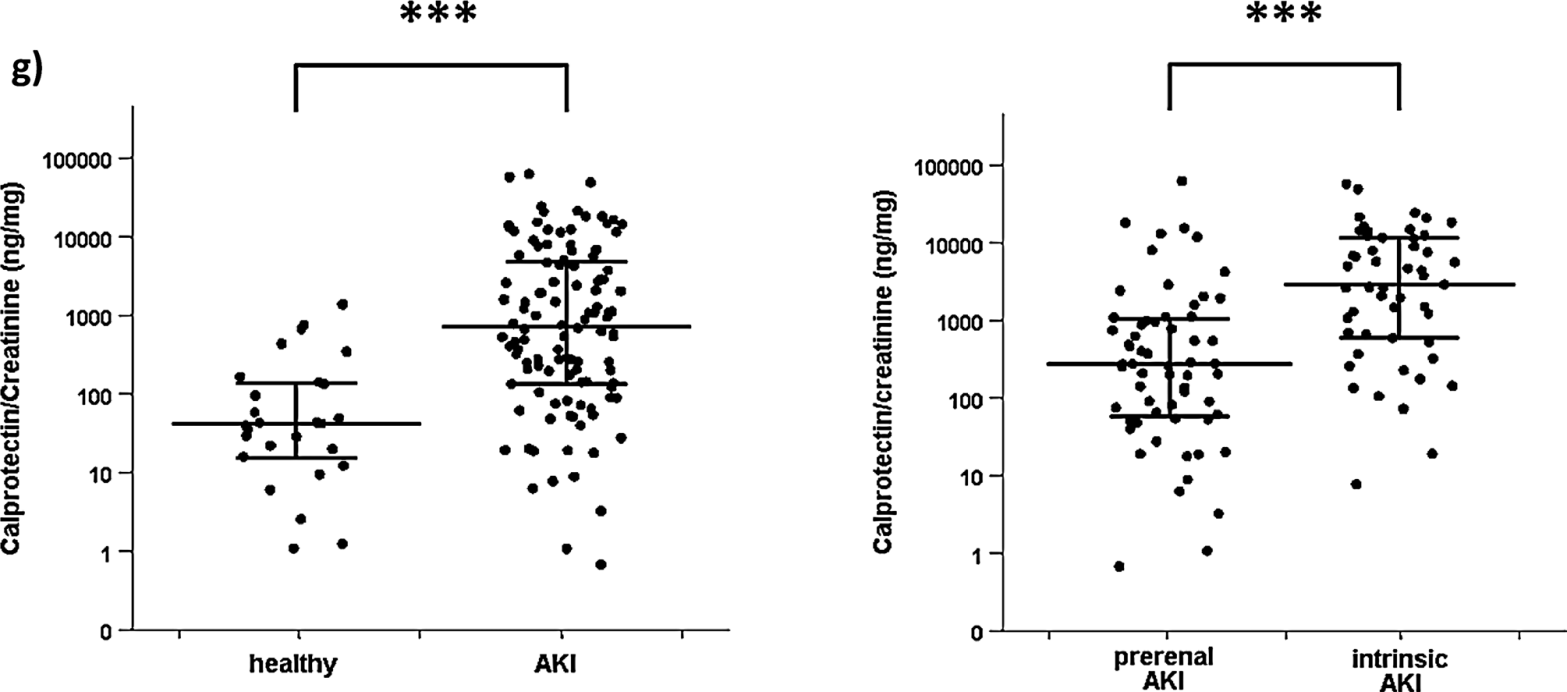
Comparison of Vanin-1/creatinine ratio between healthy persons and patients with AKI (**a**) and between patients with prerenal vs. intrinsic AKI (**b**). Comparison of diagnostic accuracy of Vanin-1/creatinine, calprotectin/creatinine, NGAL/creatinine and KIM-1/creatinine regarding diagnosis of AKI (**c**) and differentiation of prerenal from intrinsic AKI (**d**). Comparison of NGAL- (**e**), KIM-1- (**f**) and calprotectin/creatinine (**g**) Ratio between healthy persons and patients with AKI and between patients with prerenal vs. intrinsic AKI. AKI: acute kidney injury, KIM-1: Kidney injury molecule 1, NGAL: Neutrophil gelatinase-associated lipocalin, n.s.: non-significant

Median urinary Vanin-1/creatinine was significantly higher in patients with AKI than in healthy controls (1912 [812.1–4808] vs. 344.2 [165.2–553.4] pg/mg, *p* = 0.001) (Table 4). Likewise, urinary NGAL/creatinine, KIM-1/creatinine and calprotectin/creatinine were significantly higher in cases of AKI than in participants with normal renal function (*p* = 0.001). The respective AUROC for NGAL-, KIM-1- and calprotectin/creatinine were 0.92, 0.96, 0.82, whereas the urinary Vanin-1/creatinine AUROC was AUROC 0.86 (Table 4; Fig. 2). Furthermore, we conducted a subanalysis in patients with prerenal AKI to assess the effect of renin-angiotensin-aldosteron-system inhibitors (RAAS inhibitors) on urinary Vanin-1/Creatinine levels. No significant difference between patients who were on baseline RAAS inhibitors versus persons who were not was recorded (U = 356, *p* = 0.248).

**Table 4:** Urinary biomarkers

	Urinary Vanin-1/creatinine	Urinary NGAL/creatinine	Urinary KIM-1/creatinine	Urinary calprotectin/creatinine
AKI	1912 [812.1–4808] pg/mg	148.1[36.14–447.90] ng/mg	4483 [2655–8088] pg/mg	722.7 [138.6–4975] ng/mg
No AKI (healthy)	344.2 [165.2-553.4] pg/mg	7.76 [4.31–13.92] ng/mg	381.8 [228.7–799.0] pg/mg	42.0 [15.7–141.0] ng/mg
*p*	**0.001**	**0.001**	**0.001**	**0.001**
AUROC 95% CI	0.86 0.80–0.93	0.92 0.87–0.96	0.96 0.92-1.0	0.82 0.74–0.90
Sensitivity	77.7%	87.5%	91.0%	73.2%
Specificity	88.9%	85.2%	92.6%	81.4%
PPV	96.7%	96.1%	98.1%	94.3%
NPV	49.0%	62.2%	71.43%	42.3%
Cut-off (Youden index)	772.7 pg/mg	17.19 ng/mg	1540 pg/mg	170.7 ng/mg
Prerenal AKI	1587 [463.4–4303] pg/mg	57.28 [18.33–329.8] ng/mg	4393 [2245–7813] pg/mg	274.6 [58.02–104]) ng/mg
Intrinsic AKI	3042 [1149–5904] pg/mg	278.3 [108.1-801.4] ng/mg	4564 [2723–8101] pg/mg	2897 [593.7-11683] ng/mg
*p*	**0.096**	**0.001**	0.4149	**0.001**
AUC 95% CI	0.64 0.54–0.74	0.71 0.61–0.80	0.55 0.44–0.65	0.77 0.68–0.86
Sensitivity	90.2%	90.2%	49.0%	68.6%
Specificity	36.0%	49.1%	65.6%	80.3%
PPV	54.1%	59.7%	54.4%	74.5%
NPV	81.4%	85.7%	60.6%	75.4%
Cut-off (Youden index)	806.1 pg/mg	51.9 ng/mg	5530 pg/mg	1178 ng/mg

AKI: acute kidney injury; AUROC: area under the curve (ROC analysis); CI: confidence interval; KIM-1: kidney injury molecule 1; NGAL: neutrophil gelatinase-associated lipocalin; NPV: negative predictive value; PPV: positive predictive value; interquartile range [25%-75%] is presented in brackets

The diagnostic accuracy of urinary Vanin-1/creatinine in the differentiation of prerenal and intrinsic AKI was low, yielding an AUROC of 0.64 (Table 4; Fig. 2). As expected, the difference of the above ratio between the two groups did not reach statistical significance (Table 4). In comparison, AUROC for NGAL/creatinine and calprotectin/creatinine were higher (0.71 and 0.77 respectively), whereas KIM-1 showed the lowest diagnostic accuracy (0.54) among all evaluated biomarkers. No significant difference of creatinine (*p* = 0.08), GFR (*p* = 0.196), and of urinary Vanin-1/creatinine (*p* = 0.53), NGAL/creatinine (*p* = 0.28), KIM-1/creatinine (*p* = 0.37) and calprotectine/creatinine ratio (*p* = 0.99) could be recorded in patients with histopathological confirmation, compared to the subgroup where no biopsy was performed. In addition we performed a sensitivity analysis restricted to the patients where a kidney biopsy was performed. In this case the AUROC was even lower (0.43) indicating a complete loss of discriminatory capacity of the biomarker.

The above AUROCS were compared by using the online calculator provided by MedCalc and based on the method described by Hanley & McNeil [22]. The AUROC of urinary Vanin-1/creatinine ratio was not significantly different from the AUROCs of NGAL/creatinine, calprotectin/creatinine and KIM-1/creatinine (*p* = 0.31, *p* = 0.22, *p* = 0.058 respectively).

## Discussion

To our best knowledge, this is the first time where the diagnostic value of urinary Vanin-1 has been assessed in regard to its ability to distinguish prerenal from intrinsic AKI in humans. What is more, a comparison of its diagnostic performance has been compared to other biomarkers associated with AKI. Urinary Vanin-1 has emerged as a promising biomarker which could help distinguishing between prerenal and intrinsic AKI. These expectations have been based on previous studies where Vanin-1 increased early in cases of tubular damage and necrosis in animal models [18, 23, 24], in oncologic patients with drug induced renal injury [25], as well as in obstructive nephropathy [26]. However, studies assessing the diagnostic performance of Vanin-1 in regard to prerenal and intrinsic AKI differentiation have been lacking, till now.

In our study the urinary Vanin-1/creatinine ratio proved to be significantly higher in patients with AKI compared to healthy controls and the respective AUROC reached a relative high 0.86. In comparison, in a previous study evaluating two cell cycle arrest biomarkers, urinary insulin-like growth factor binding protein (IGFBP) 7 and tissue inhibitor of metalloproteinase (TIMP)-2, as possible predictors of AKI, the recorded AUCs were 0.77 and 0.75, respectively [27]. Similarly, Bichar et al. reported an AUC of 0.82 of a single urinary [TIMP-2]·[IGFBP7] test for predicting severe AKI in the following 12 h [28]. However, external validation and verification of these findings in larger, heterogeneous cohorts is pending. Besides, differentiating between prerenal and intrinsic AKI, biomarkers have been utilized in a greater frame of clinical scenarios. As Coca et al. demonstrated, early urinary biomarkers can reliably predict AKI duration in postoperative patients, distinguishing between persistent versus transient AKI [29]. In the same study ≥ 7-day AKI was strongly associated with 3-year mortality. In our study we focused solely on the discriminative capacity of urinary Vanin-1/Creatinine ratio in regard to differentiation of prerenal from intrinsic AKI. Only patients with stage II or III were eligible which means that in our case biomarkers were assessed only after an AKI was diagnosed and that AKI could have been present for some time. Studies with a longer follow-up time are needed to evaluate any prognostic value of urinary Vanin-1/Creatinine ratio concerning the AKI duration, the risk of progression to AKD and CKD and mortality.

AKI is a clinical entity associated with increased morbidity and mortality in hospitalized patients. Prompt and appropriate treatment is crucial before a, usually, transitory kidney dysfunction progresses into a permanent decline of the renal function. Hence, the importance of distinguishing between prerenal, intrinsic and postrenal AKI cannot be overstated. The gold standard method to distinguish prerenal from intrinsic AKI is the kidney biopsy and histological examination. However, in practice, this invasive procedure is associated with a not-negligible risk of complications and is preserved only for cases where no improvement of renal function through volume repletion is achieved and where no apparent cause of AKI in the patient’s history and current medical condition can be identified. Consequently, the use of clinical algorithms like the one we chose in this study remains a common way to distinguish between prerenal and intrinsic AKI. However, the specificity of these methods has certain limits and false classification of prerenal as intrinsic AKI or the opposite cannot be excluded. In an effort to increase the diagnostic accuracy, a series of methods based on urine chemistries and urine analysis have been proposed in the past. The fractional excretion of sodium (FENa) [30] and fractional excretion of urea (FEUrea) [31] are among the oldest and best studied diagnostic methods, relying on urine chemistry, used to differentiate prerenal from intrinsic AKI. Simple and easy to obtain, FENa shows a high sensitivity and specificity in differentiating between prerenal and intrinsic AKI, nonetheless it performs adequately only in oliguric patients without CKD and not on diuretic treatment [32]. FEUrea which has been proposed as an alternative urine marker to FENa in patients on diuretics or in cases of pre-existing chronic kidney disease showed similar poor performance [32–34]. As to the urine microscopy, although readily available and inexpensive, it requires certain training and expertise and is time-consuming [35]. Novel, promising biomarkers like NGAL, KIM-1, and L-FABP have been proposed over the past years in an attempt to overcome the caveats of traditional diagnostic methods like FENa, FEUrea and urine microscopy. Studies have demonstrated that these biomarkers are increased in cases of ischemic tubular damage, thus are not prone to disruption by the use of diuretics [36–38]. Nevertheless, despite the evidence supporting the application of these parameters in the clinical practice, significant knowledge gaps still remain which warrant further research in the field [39].

Our study has limitations. Firstly, its cross-sectional character limits the strength of its findings. Second, the categorization of the AKI as prerenal or intrinsic has been based, in the majority of the patients, on a clinical algorithm whereas kidney biopsy, the gold standard for diagnosing intrinsic AKI was performed only in a fraction of the patients. As a result we must acknowledge that the classification into intrinsic AKI relied, in most patients, on clinical inference and carried a risk of misclassification. Notably, the observed loss of diagnostic capacity of the urinary Vanin-1/Creatinine ratio in the subgroup of patients where the diagnosis was confirmed with biopsy, indicates that the relatively higher AUROC recorded in the full cohort could be due to misclassification bias in the majority of cases where the clinical algorithm was applied for AKI diagnosis. Moreover, the inverse AUC (< 0.5) in the biopsy subgroup further suggests that the way of association may differ when cause of AKI is confirmed with biopsy, thus emphasizing the diagnostic value of the gold standard method. Making matters worse, in many patients, the same causative factor, for example decreased renal perfusion, can result in a combination of prerenal and intrinsic AKI. Another important limitation is the relative wide time-span for urine sampling (3days) relative to AKI-onset set in our study. These traits could significantly distort the results of the study. Moreover, the single-centre character of the study does not allow the generalization of the previous findings. In addition, the modest size, especially compared to other relevant multicentre studies evaluating AKI-biomarkers [40–42], results in wide confidence intervals. Thus, differences between AUROCs should be interpreted cautiously. It must be mentioned that the healthy persons group was significantly younger than our study population. Nonetheless, the purpose of the control group in this study was having a population of healthy patients without any evidence or history of CKD, that could serve as a comparison for the urine samples and the urinary biomarkers (see Table 4) taken from patients with AKI. As a consequence, a younger population in the control group was expected, taking into account the gradual decline of kidney function in older persons. Larger, multicentre studies with the involvement of a bigger proportion of patients, who underwent kidney biopsy, could possibly shed light and strengthen the above findings.

In conclusion, in this multicentre study the urinary Vanin-1/creatinine ratio failed to provide any information regarding the type of AKI. Moreover, its diagnostic performance was poor in distinguishing prerenal from intrinsic AKI. However, given the fact that urinary Vanin-1/creatinine ratio was increased in patients with AKI compared to healthy controls and the previous studies which have demonstrated the multiple clinical applications urinary biomarkers can have, further larger studies which assess the prognostic value of urinary Vanin-1/creatinine ratio in similar context are justified.

## Data Availability

The datasets generated during and analysed during the current study are available from the corresponding author on reasonable request.
